# A Shorter Equilibration Period Improves Post-Warming Outcomes after Vitrification and in Straw Dilution of In Vitro-Produced Bovine Embryos

**DOI:** 10.3390/biology10020142

**Published:** 2021-02-10

**Authors:** Iris Martínez-Rodero, Tania García-Martínez, Erika Alina Ordóñez-León, Meritxell Vendrell-Flotats, Carlos Olegario Hidalgo, Joseba Esmoris, Xabier Mendibil, Sabino Azcarate, Manel López-Béjar, Marc Yeste, Teresa Mogas

**Affiliations:** 1 Department of Animal Medicine and Surgery, Autonomous University of Barcelona, ES-08193 Cerdanyola del Vallès, Spain; iris.martinez@outlook.com (I.M.-R.); taniagarciamartinez@gmail.com (T.G.-M.); alina.mvzalina@gmail.com (E.A.O.-L.); meritxell.vflotats@gmail.com (M.V.-F.); 2 Grupo InVitro, Villahermosa MX-86040, Mexico; 3 Department of Animal Health and Anatomy, Autonomous University of Barcelona, ES-08193 Cerdanyola del Vallès, Spain; manel.lopez.bejar@uab.cat; 4 Department of Animal Selection and Reproduction, The Regional Agri-Food Research and Development Service of Asturias (SERIDA), ES-33394 Gijón, Spain; cohidalgo@serida.org; 5 Tekniker, Basque Research and Technology Alliance (BRTA), ES-20600 Eibar, Spain; joseba.esmoris@tekniker.es (J.E.); xabier.mendibil@tekniker.es (X.M.); sabino.azcarate@tekniker.es (S.A.); 6 Department of Biology, Institute of Food and Agricultural Technology, University of Girona, ES-17004 Girona, Spain; marc.yeste@udg.edu

**Keywords:** cow, cryopreservation, expanded blastocyst, total cell number, inner cell mass, trophectoderm, SOX2, TUNEL, apoptosis, gene expression

## Abstract

**Simple Summary:**

For more productive and sustainable livestock activity, various reproductive biotechnologies are being incorporated into breeding programs to accelerate genetic improvement. Among these strategies, embryo cryopreservation is a key technique for the conservation and dissemination of genetic resources while also optimizing animal production and biosafety. Though vitrification techniques are rapidly gaining acceptance due to their speed, simplicity, and feasibility, their practical applications in veterinary reproduction are limited because there is no standard protocol that facilitates warming in field conditions. Moreover, working time increases when a large number of embryos has to be cryopreserved. In-straw warming/dilution methods allow for the vitrification of embryos and their direct transfer to the uterus of recipients. In order to increase vitrification efficiency by reducing the working time and simplifying warming in field conditions, in vitro-derived cattle embryos at the expanded blastocyst stage were vitrified by using two different protocols (short equilibration vitrification and long equilibration vitrification) and in straw diluted/warmed. The short equilibration protocol improved vitrification outcomes in terms of embryo survival and hatching ability, and it improved embryo quality in terms of higher total cell number and lower apoptosis rate. A gene expression analysis of surviving embryos also indicated that the short equilibration treatment could lead to the production of more high-quality blastocysts.

**Abstract:**

This study was designed to the optimize vitrification and in-straw warming protocol of in vitro-produced bovine embryos by comparing two different equilibration periods, short equilibrium (SE: 3 min) and long equilibrium (LE: 12 min). Outcomes recorded in vitrified day seven (D7) and day eight (D8) expanded blastocysts were survival and hatching rates, cell counts, apoptosis rate, and gene expression. While survival rates at 3 and 24 h post-warming were reduced (*p* < 0.05) after vitrification, the hatching rates of D7 embryos vitrified after SE were similar to the rates recorded in fresh non-vitrified blastocysts. The hatching rates of vitrified D8 blastocysts were lower (*p* < 0.05) than of fresh controls regardless of treatment. Total cell count, and inner cell mass and trophectoderm cell counts were similar in hatched D7 blastocysts vitrified after SE and fresh blastocysts, while vitrified D8 blastocysts yielded lower values regardless of treatment. The apoptosis rate was significantly higher in both treatment groups compared to fresh controls, although rates were lower for SE than LE. No differences emerged in *BAX*, *AQP3*, *CX43,* and *IFNτ* gene expression between the treatments, whereas a significantly greater abundance of *BCL2L1* and *SOD1* transcripts was observed in blastocysts vitrified after SE. A shorter equilibration vitrification protocol was found to improve post-warming outcomes and time efficiency after in-straw warming/dilution.

## 1. Introduction

In beef and dairy cattle, in vitro embryo production (IVP) through assisted reproductive technologies is gaining popularity as an alternative to artificial insemination and in vivo embryo transfer to improve genetic gains. This approach also helps circumvent breeding problems such as cows that may not ovulate or show compromised fertility during periods of heat stress (reviewed by [1]). Because of the large numbers of embryos generated through in vitro technologies, the cryopreservation of these embryos has become an important topic of research. Studies have shown that the in vivo-derived transferable-stage embryos of many mammalian species can be successfully preserved through conventional slow freezing. In contrast, vitrification seems the most effective method for embryos produced in vitro, as they are highly susceptible to cryoinjury [2]. While vitrification is simpler, faster, and cheaper than slow cryopreservation methods, it requires higher concentrations of cryoprotectant agents (CPAs), which could have deleterious effects on embryo development after their warming. To minimize this effect, warming is achieved via a complex dilution procedure along with the use of a stereomicroscope to completely remove the vitrification solution. When working under farm conditions, this procedure is especially technically demanding. 

When using vitrification technology in veterinary practice, a practical approach is needed for the warming of vitrified embryos so that embryos can be directly and easily transferred to the uterus. Thus far, there have been several attempts to replace successive dilution steps with one-step in-straw cryoprotectant dilution [3,4,5,6,7,8,9,10,11,12,13,14,15] However, in some of these procedures, in-straw embryo warming requires more than one dilution step and the proper handling of the carrier system, thus demanding more accuracy when these techniques are to be used in the field by embryo-transfer practitioners [7,9,10,12]. Using the VitTrans device designed by our group, IVP embryos are easily warmed/diluted in-straw for their transfer to recipient females in field conditions [16]. The performance of the VitTrans device assessed in terms of post-warming survival rates after 24 h of the culture of IVP bovine embryos is comparable to that observed with our control vitrification-warming method [16].

For the vitrification of a solution, a radical increase in both the cooling rate and cryoprotectant concentration is required. Unfortunately, most cryoprotectants have some negative effects, including toxicity and osmotic injury. Though there is no consensus regarding the toxicity of penetrating CPAs, it is widely accepted that the higher their concentration and exposure temperature, the greater their toxicity. Hence, any variation in exposure time prior to cooling can cause dramatic differences in cellular hydration [17,18]. In any vitrification protocol it is accordingly important to achieve an adequate balance between obtaining a high level of dehydration and high viscosity while also avoiding toxicity. The first step is usually an equilibration stage in a solution containing a relatively low CPA concentration, followed by ultra-short (30–90 s) exposure to a vitrification medium with a higher concentration of cryoprotectant (usually double the initial concentration) and dehydrating agent (usually a disaccharide, such as sucrose). Exposure to the equilibration medium may be short (e.g., 1 min followed by 25 s during vitrification) [12,19], last for 3min followed by vitrification for 25–30 s [9,15,20,21], or be even longer (e.g., 10–15 min followed by vitrification for 60 s) [22]. These durations have provided adequate blastocyst survival, blastocyst hatching, and pregnancy rates. 

Given this background, we hypothesized that a shorter exposure time in just the first step of the in-straw method protocol could be a safe approach to allow for the delivery of the cryoprotectants to the blastocyst, thereby minimizing the likelihood of toxicity or osmotic damage. The objective of the present study was to determine whether a first equilibration step of the vitrification protocol shortened from 12 to 3 min would serve to improve the quality of vitrified/in straw-warmed day seven- and day eight-expanded blastocysts. Outcomes were assessed in the warmed embryos in terms of survival rates, differential cell counts, cell apoptosis, and relative abundances of mRNAs of genes with a role in apoptosis, oxidative-stress, water channels, gap junctions, and implantation. 

## 2. Materials and Methods

### 2.1. Chemicals and Suppliers

Unless stated differently, chemicals and reagents were purchased from Sigma-Aldrich (Sant Louis, MO, USA).

### 2.2. In Vitro Production of Bovine Blastocysts 

Embryos were produced according to our previously established procedures [23], with minor modifications. In brief, cumulus oocyte complexes (COCs) were harvested by aspiration from 3- to 8-mm follicles from cow ovaries previously obtained from a slaughterhouse and transferred to the laboratory in a saline solution (0.9% NaCl) at 36.5 °C. COCs were washed three times in modified Dulbecco’s phosphate-buffered saline (PBS) containing 0.5 mg/mL of bovine serum albumin (BSA), 36 mg/mL of pyruvate, and 50 mg/mL of gentamicin. For in vitro maturation (IVM), 40–50 COCs with three or more cumulus cells layers showing a homogeneous cytoplasm were placed in 500 µL of a maturation medium in four-well plates and cultured for 24 h at 38.5 °C in 5% CO_2_ humidified air. The IVM medium was a tissue culture medium (TCM-199) supplemented with 10% (*v*/*v*) fetal calf serum (FCS), 10 ng/mL of epidermal growth factor, and 50 mg/mL of gentamicin. 

For in vitro fertilization (IVF), the thawed sperm of a fertile bull was used to obtain motile spermatozoa. After its centrifugation for 10 min at 300× *g* on a gradient consisting of 1 mL 40% BoviPure on 1 mL of 80% BoviPure (Nidacon International AB, Mölndal, Göthenburg, Sweden), the resulting sperm fraction was resuspended in 3 mL of BoviWash (Nidacon International AB, Mölndal, Göthenburg, Sweden) and was again pelleted by centrifugation for 5 min at 300× *g*. Spermatozoa were then counted and diluted in a proper volume of a fertilization medium (Tyrode’s medium supplemented with 6 mg/mL fatty acid-free BSA, 22 mM Na-lactate, 25 mM bicarbonate, 1 mM Na-pyruvate, and 10 mg/mL of heparin–sodium salt) to give a final spermatozoa concentration of 2 × 10^6^/mL. In vitro matured oocytes (40–50) in 250 µL of the fertilization medium were co-incubated at 38.5 °C in a 5% CO_2_ humidified air with 250 µL of sperm suspension to give a final concentration of 1 × 10^6^/mL of spermatozoa. 

After 18 h post-insemination (hpi), presumptive zygotes were gently pipetted in PBS to denude them and transferred to 25-µL drops of the culture medium (1 embryo/µL) covered by 3.5 mL of mineral oil. The culture medium was synthetic oviductal fluid (Caisson Labs, Smithfield, UT, USA) containing 0.96 μg/mL of BSA, 2% (*v*/*v*) FCS, 88.6 μg/mL of Na-pyruvate, 2% (*v*/*v*) non-essential amino acids, 1% (*v*/*v*) essential amino acids, and 0.5% (*v*/*v*) gentamicin. Plates with the zygotes were incubated either for 7 or 8 days at 38 °C in a 5% CO_2_/5% O_2_/90% N_2_ humidified air. The recorded outcomes were the cleavage rate at 48 hpi and the number of blastocysts on days 7 and 8 after insemination. 

### 2.3. Embryo Vitrification and Warming 

**Figure 1:**
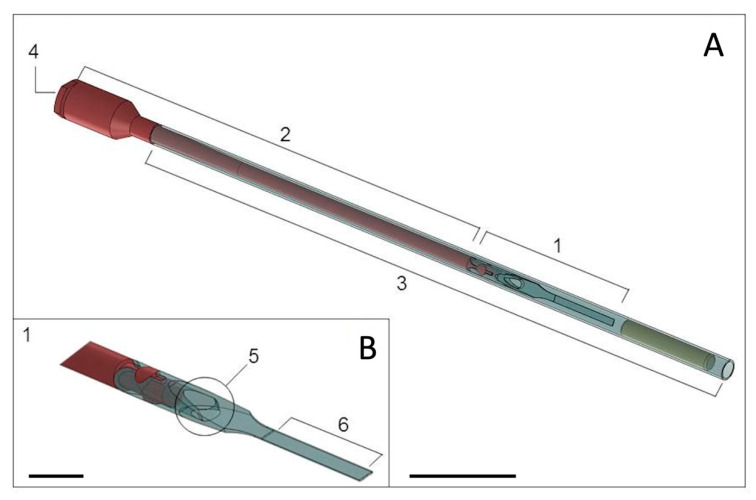
(**A**) The VitTrans device comprises a carrier where the embryo is loaded (1), a hard plastic handle with an inner channel (2) into which warming solutions are introduced to dilute the cryoprotectant and transport the embryo to the straw (3) for transfer, and a Luer syringe connector (4) to connect the device to the warming solution source. The straw (3) acts as a cover to protect the device from mechanical damage during storage. During warming, it serves as a 0.5 mL straw for sample dilution and direct embryo transfer. Scale bar: 2 cm. (**B**) Closer view of the end of the device (1) showing the outflow of the inner channel (5) and embryo attachment piece (6). Scale bar: 1 cm.

Blastocysts were vitrified using the VitTrans device and vitrification-warming solutions, as previously described by Morató and Mogas [16]. The VitTrans device is composed of a plastic carrier where the embryo is loaded, a hard plastic handle to hold the device, and a covering straw that protects the device from mechanical damage during storage and serves as a 0.5 mL straw for embryo dilution during warming and direct transfer. The handle has a Luer syringe connector and an inner channel through which the warming solution is introduced to dilute the cryoprotectants, detach the embryo from the carrier, and displace the embryo to the straw for transfer (Figure 1). The holding medium (HM) used to formulate the vitrification-warming solutions was TCM-199 HEPES supplemented with 20% (*v*/*v*) FCS. These procedures were performed under a laminar flow hood using a heated surface at 38.5 °C and a stereomicroscope to visualize each step.

#### 2.3.1. Vitrification Protocol

Day 7 (D7) and day 8 (D8) blastocysts were randomly transferred to an equilibration solution (ES) containing 7.5% (*v*/*v*) ethylene glycol (EG) and 7.5% (*v*/*v*) dimethyl sulfoxide (DMSO) in the HM for 3 min (short equilibration: SE) or 12 min (long equilibration: LE). The blastocysts were then transferred to a vitrification solution consisting of 15% (*v*/*v*) EG, 15% (*v*/*v*) DMSO, and 0.5 M sucrose dissolved in the HM. After incubating for 30–40 s, embryos (up to 2) were loaded onto the embryo attachment piece of the VitTrans device. Then, most of the solution was removed, leaving only a thin layer on the blastocysts, and the sample was quickly plunged in liquid nitrogen. Subsequently, the VitTrans device was covered with the 0.5 mL plastic straw. The entire process from immersion in the vitrification solution to plunging in liquid nitrogen took less than 1 min. The devices were stored in liquid nitrogen until further use. 

#### 2.3.2. Warming Protocol

For warming, the cover of the VitTrans device was twisted for 10 s inside liquid nitrogen to release pressure. Then, the whole VitTrans device (with its cover) was removed from the liquid nitrogen, held for 1 s in the air, and submerged in a water bath at 45 °C for 3 s, leaving the hard handle above the water surface. While in the water bath, a syringe containing the diluting solution (0.5 M sucrose in the HM) at 45 °C was connected to the hard handle using the Luer connector. Next, the whole VitTrans device (with its cover) was removed from the water bath as the diluting solution was injected through the lumen of the device. Once the diluting solution entered the straw, the outside was dried to remove any remaining water, and the VitTrans device was removed from the straw. At this point, the straw containing the warmed embryo was ready for transfer.

To determine embryo survival in subsequent experiments, the cotton plug end of the straw was pushed, and the contents of the straw was expelled into a Petri dish. Blastocysts were then transferred to the culture medium and incubated for 24 h at 38 °C in a 5% CO_2_/5% O_2_/90% N_2_ humidified air. Survival rates were expressed as proportions of blastocysts showing signs of re-expansion at 3 and 24 h post-warming. Hatching rates were defined as the proportions of hatching/hatched blastocysts at 24 h post-warming (experiment 1; 9 replicates). Fresh non-vitrified D7 or D8 blastocysts served as non-vitrified controls. The expanded and hatching/hatched blastocysts from groups D7-Control, D7-SE, D7-LE, D8-Control, D8-SE, and D8-LE were fixed and immunostained to determine the variables: total cell number (TCN), inner cell mass (ICM) cell number, trophectoderm (TE) cell number, and apoptosis rate (AR) (experiment 2; 4 replicates). Surviving expanded and hatching/hatched blastocysts from D7-Control, D7-SE, and D7-LE were collected in pools of 5 blastocysts, snap frozen in liquid nitrogen, and stored at −80 °C until RNA isolation and RT-qPCR analysis were conducted (experiment 3; 5 replicates).

### 2.4. Differential Staining and TUNEL

**Figure 2:**
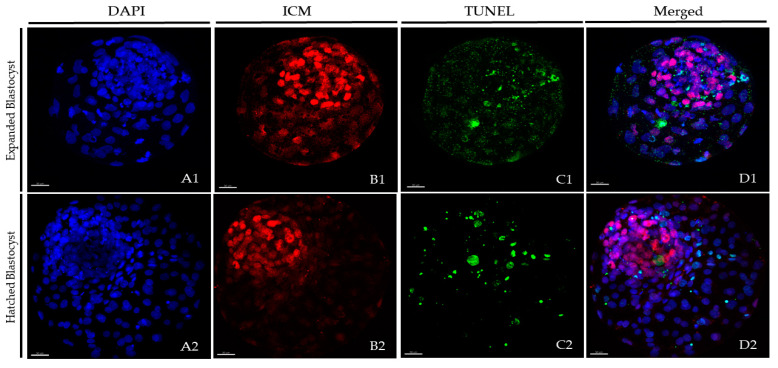
Representative pictures of post-warmed expanded and hatched blastocysts vitrified after short or long equilibration in the VitTrans procedure. After 24 h of culture post-warming, blastocysts were subjected to the TUNEL (terminal deoxynucleotidyl transferase dUTP nick end labelling) technique combined with differential staining. DAPI (4′,6-diamidino-2-phenylindole) (blue), SOX2 (red), and TUNEL (green) staining were examined using DAPI, SOX2-Alexa Fluor 568, and FITC filters, respectively, for total (**A1**,**A2**), inner cell mass (ICM) (**B1**,**B2**), and apoptotic (**C1**,**C2**) cell counts. An overlay is provided in (**D1**,**D2)**. (**A1**,**B1**,**C1**,**D1**) Expanded blastocysts; (**A2**,**B2**,**C2**,**D2**) hatched blastocysts. Scale bar: 30 μm.

At 24 h post-warming, expanded and hatched blastocysts surviving vitrification in each group underwent immunostaining plus the TUNEL (terminal deoxynucleotidyl transferase dUTP nick end labelling) assay to quantify TCN, ICM cell number, TE cell number, and AR. Fresh non-vitrified D8 blastocysts served as controls. The protocol for embryo staining was based on the work of Vendrell-Flotats et al. [24] with some modifications. Unless stated otherwise, all steps were performed at 38.5 °C. Briefly, the fixation of blastocysts was done in 2% (*v*/*v*) paraformaldehyde diluted in PBS for 15 min at room temperature. Fixed embryos were washed three times in PBS for 5 min and then permeabilized in 0.01% (*v*/*v*) Triton X-100 diluted in PBS containing 5% (*v*/*v*) normal donkey serum (PBS-NDS) for 1 h at room temperature. After permeabilization, the embryos were washed in PBS (3×) for 5 min and incubated overnight with a mouse anti-SOX2 primary antibody (1:100; Invitrogen, Carlsbad, CA, USA) at 4 °C in a humidified chamber. Next, the embryos were washed with 0.005% (*v*/*v*) Triton X-100 diluted in PBS-NDS for 20 min and incubated with a goat anti-mouse IgG Alexa Fluor 568 secondary antibody (1:500; Thermo Fisher, Waltham, MA, USA) for 1 h in a humidified chamber. Next, they were placed in 0.005% (*v*/*v*) Triton X-100 diluted in PBS-NDS for 20 min, washed in PBS (3×) for 5 min, and incubated for 1h in the dark in the TUNEL reaction mixture dilution according to manufacturer’s instructions (1:10; In Situ Cell Death Detection Kit, Fluorescein). In each assay, blastocysts for positive and negative controls were included. Positive controls consisted of blastocysts exposed to DNase I for 15 min, and negative controls consisted of blastocysts not exposed to the terminal TdT enzyme. After TUNEL incubation, embryos were washed in 0.005% (*v*/*v*) Triton X-100 diluted in PBS-NDS for 5 min, mounted on coverslips that had been pretreated with poly-l-lysine within a drop (3-µL) of Vectashield, which contained 125 ng/mL of 4′,6-diamidino-2-phenylindole (DAPI) (Vectorlabs, Burlingame, CA, USA). A slide was used to flatten the preparation, which was sealed with nail varnish and kept at 4 °C in the dark until examination within the next 3 days. Confocal images of 0.5-µm serial sections were taken with a confocal laser scanning microscope (Leica TCS SP5, Leica Microsystems, Wetzlar, Hesse, Germany) to examine the ICM cell nuclei (SOX2-Alexa Fluor 568; excitation 561 nm), cell nucleus (DAPI; excitation 405 nm), and DNA fragmentation (fluorescein isothiocyanate-conjugated TUNEL label; excitation 488 nm). Images captured with confocal microscope were analyzed using the Imaris 9.2 software (Oxford Instruments, Abingdon-on-Thames, Oxfordshire, UK) to determine TCN, ICM cell number, and apoptotic cells. Individual nuclei were counted as TE cells (SOX2(−); blue stain) or ICM cells (SOX2(+); red stain) and intact (TUNEL(−); blue/red stain) or fragmented (TUNEL(+), green stain) DNA (Figure 2). The total number of cells was calculated as the sum of the TE and ICM cells. The AR was calculated as the ratio of TUNEL(+) cells/total number of cells.

### 2.5. RNA Extraction and Relative Quantification of mRNA by Real-Time Reverse Transcription PCR 

Twenty four hours after warming, the gene expression in the blastocysts was analyzed through RNA extraction and RT-qPCR as described elsewhere [25]. Warmed blastocysts were prepared for this analysis by washing three times in Dulbecco’s PBS containing 0.3% (*w*/*v*) polyvinyl alcohol (PVA) at 38.5 °C, pipetting pools of 5 embryos from 4 independent replicates into 0.5 mL microtubes and plunging the microtubes in liquid nitrogen for storage at −80 °C until analysis.

For poly-(A)-RNA extraction, the Dynabeads mRNA Direct Extraction Kit (Thermo Fisher, Waltham, MA, USA) was used according to the manufacturer’s instructions but with slight modifications. Blastocyst pools were lysed in 50 μL of a lysis buffer for 5 min at room temperature while gently pipetting. Next, the lysate was hybridized with 10 μL of prewashed beads for 5 min at room temperature by gentle shaking. The poly-(A)-RNA-bead complexes were then washed at room temperature twice in 50 μL of washing buffer A and twice again in 50 μL of washing buffer B. Complexes were eluted in 16 μL of Tris HCl and then heated to 70 °C for 5 min to open the helix. For subsequent reverse transcription (RT), the extracted poly-(A)-RNA was mixed with 4 μL of qScript cDNAsupermix (Quanta Biosciences, Gaithersburg, MD, USA) containing random primers, dNTPs, oligo-dT primers, and qScript reverse transcriptase. The RT reaction consisted of a first step of 5 min at 25 °C for DNA, followed by 1 h at 42 °C for the RT of mRNA, and 10 min at 70 °C for reverse transcriptase enzyme denaturing. The resultant cDNA was diluted in 25 μL of Tris HCl. 

To quantify the relative abundance of mRNA transcripts, the qPCR method was conducted in a 7500 Real Time PCR System (Applied Biosystems, Foster City, CA, USA). The reaction mixture consisted of 10 μL of Fast SYBR Green Master Mix (Thermo Fisher, Waltham, MA, USA), 1.2 μL of each primer (300 nM; Thermo Fisher, Waltham, MA, USA), and 2 μL of the cDNA template. For a final volume of 20 μL, nuclease-free water was added. The PCR amplification procedure was as follows: one denaturation cycle at 95 °C for 10 min, 45 amplification cycles and a denaturation step at 95 °C for 15 s, an annealing step at 60 °C for 1 min, and a final extension at 72 °C for 40 s. Fluorescence data were obtained during this final extension step. The nature of the amplified PCR product was checked through melting curve analysis and gel electrophoresis (2% agarose gel which contained 0.1 μg/mL SafeView; Applied Biological Materials, Vancouver, British Columbia, Canada). To prepare this curve, samples were heated from 50 to 95 °C, and each temperature was held for 5 s while monitoring fluorescence. Three technical replicates of each of the four biological replicates were run per individual gene. To check for possible cross-contamination, negative controls for the template and reverse transcription were run in each assay. 

To measure the relative expression of six candidate genes (*BAX*, *BCL2L1*, *AQP3*, *SOD1*, *CX43*, and *IFNτ*) in vitrified/warmed viable blastocysts 24 h after warming, the comparative threshold cycle (Ct) method was employed, using the housekeeping (HK) genes *PPIA* and *H3F3A* as normalizers. To determine the threshold cycle for each sample, after each elongation step, fluorescence data were obtained. The threshold cycle set in the log-linear phase indicates the PCR cycle number for which the fluorescence generated was just above background fluorescence. Within this amplification curve region, a difference of one cycle equated to PCR product doubling. To calculate ΔCt values, the mean *PPIA* and *H3F3A* Ct values for each sample were subtracted from the Ct value of each target gene separately for each replicate. To calculate ΔΔCt, the ΔCt value was subtracted from the average ΔCt for all embryos per target. Using the formula 2−(ΔΔCt), fold differences in relative transcript abundances were estimated for target genes, assuming an amplification efficiency of 100%. Table 1 provides the primer sequences used to amplify each gene, along with their corresponding amplicon sizes and GenBank accession numbers. As expected, controls lacking templates were not amplified or returned a Ct that was 10 points higher than the average Ct for the genes. This analysis was conducted independently as four replicate experiments.

### 2.6. Statistical Analysis

The statistical package IBM SPSS Version 25.0 (IBM Corp., Chicago, IL, USA) was used to perform all statistical tests. First, the normality of the data was checked using the Shapiro-Wilk test, and homogeneity of variances using the Levene test.

Survival rates were compared by a two-way ANOVA followed by Bonferroni test for pair-wise comparisons. Total cell counts, number of ICM cells, and apoptosis rate were analyzed by a three-factor general linear model. Relative transcript abundances were assessed by the two-factor ANOVA followed by the post-hoc Bonferroni test. Data were linearly transformed into arcsine square roots, square roots, or logarithms when data were not normally distributed or variances were not homogenous. When transformed data did not fulfil parametric assumptions, Kruskal–Wallis and Mann–Whitney tests were used as non-parametric alternatives. Data are expressed as means ± standard error of the mean (SEM). Significance was set at *p* ≤ 0.05.

## 3. Results

### 3.1. A Shorter Time of Exposure of Embryos to the Equilibrium Solution Leads to Improved Embryo Development (Experiment 1)

The post-warming survival and hatching rates of D7 and D8 expanded blastocysts vitrified after a short (3 min) or long (12 min) period of exposure to the equilibration solution are shown in Table 2. Vitrification led to significant reductions in D7 and D8 embryo survival rates recorded at 3 or 24 h post-warming when compared to fresh control blastocysts. While no effects of equilibration time were observed on embryo survival assessed at 3 h post-warming, both D7 and D8 vitrified blastocysts subjected to SE showed significantly higher survival and hatching rates (*p* < 0.05) than the blastocysts that were vitrified after a longer equilibration period. The hatching rates of D7 blastocysts in the SE group did not differ from those observed for the fresh non-vitrified blastocysts (31.4 ± 3.7 vs. 35.9 ± 4.0, respectively). However, vitrified D8 blastocysts showed significantly lower hatching rates than those derived from fresh non-vitrified embryos, regardless of SE or LE.

In addition, survival at 24 h post-warming was significantly higher for the vitrified D7 blastocysts than D8 blastocysts, regardless of the equilibration period. The SE treatment significantly increased the hatching capacity of vitrified D7 blastocysts when compared to vitrified D8 blastocysts. However, no differences in hatching rates were observed between D7 and D8 blastocysts vitrified after the LE treatment. 

### 3.2. Different Exposure Times to the Equilibration Solution Modify TCN, ICM Cell Numbers, TE Cell Numbers and Apoptosis Rates at 24 h Post-Warming (Experiment 2)

The outcomes TCN, ICM, and TE cell numbers, as well as AR determined 24 h post-warming of D7 and D8 expanded bovine blastocysts vitrified after the short and long equilibration times are shown in Table 3. TCN and TE cell numbers were significantly lower in expanded blastocysts derived from vitrified/warmed D7 blastocysts compared to those derived from fresh control blastocysts, regardless of the length of exposure to the equilibration solution. However, both outcome measures were similar in non-vitrified fresh and SE-vitrified D7 blastocysts reaching the hatching stage at 24 h post-warming, while they were significantly lower in LE-vitrified D7 blastocysts. The rate of apoptotic cells was significantly higher in both vitrification groups when compared to fresh controls, although vitrification using the SE protocol produced less apoptosis than when the LE protocol was used. 

No differences were observed when TCN and TE cell number were assessed at 24 h post-warming in expanded blastocysts derived from fresh D8 blastocysts or D8 blastocysts vitrified using the SE protocol. However, both counts were significantly lower in expanded blastocysts derived from D8 blastocysts vitrified using the LE protocol. ICM cell numbers in expanded blastocysts derived from vitrified/warmed D8 blastocysts were significantly lower compared to control fresh blastocysts, regardless of the vitrification protocol. A similar trend was observed for TCN, ICM, and TE cell numbers assessed in hatched blastocysts derived from vitrified/warmed D8 blastocysts. Apoptosis rates were significantly higher for vitrified/warmed D8 blastocysts when compared to non-vitrified embryos, although the SE protocol yielded a significantly lower apoptosis rate than the LE protocol.

### 3.3. Different Times of Exposure to the Equilibration Solution Modify Gene Expression Patterns in Warmed Expanded Blastocysts Vitrified Using the VitTrans as the Cryodevice (Experiment 3)

**Figure 3:**
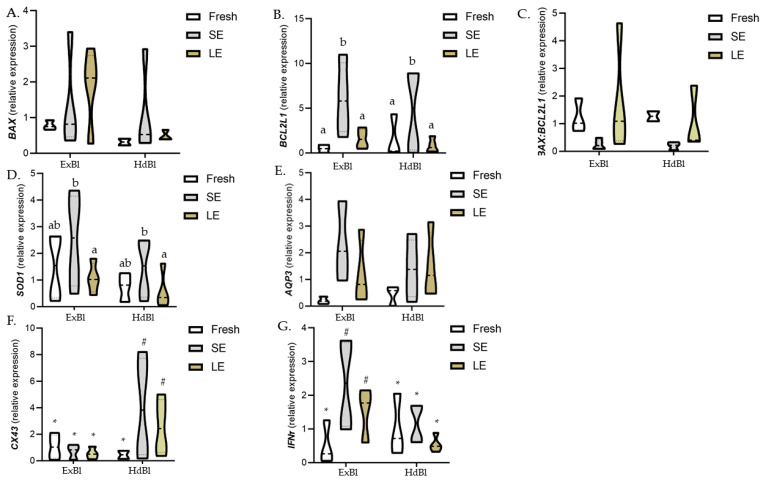
Violin plots (solid line indicates the 50% quantile) showing the expression levels of selected genes in post-warmed, expanded, and hatched blastocysts derived from D7 expanded bovine blastocysts vitrified after a short or long time of exposure to the equilibration solution. Different letters indicate significant differences between treatments (*p* < 0.05), and different symbols indicate differences between developmental stages for each specific treatment. *BAX*, BCL2 associated X apoptosis regulator; *BCL2L1*, BCL2 like 1; *SOD1*, superoxide dismutase 1; *AQP3*, aquaporin 3; *CX43*, connexin 43; *IFNτ*, interferon tau; ExBl, expanded blastocysts; HdBl, hatching/hatched blastocysts; Control: fresh non-vitrified expanded blastocysts; SE: D7 expanded blastocysts vitrified after a short equilibration time (3 min); LE: D7 expanded blastocysts vitrified after a long equilibration time (12 min).

Given the effects on embryo development and embryo quality observed in our initial experiments, the effects of the shorter and longer equilibration times on the relative abundance of genes were only assessed in post-warmed expanded and hatched blastocysts derived from vitrified/warmed D7 expanded embryos (Figure 3). While no significant differences in *BAX* expression was observed between the two treatments, the *BCL2L1* gene was overexpressed in both expanded and hatched blastocysts derived from SE-vitrified blastocysts compared to blastocysts derived from fresh or LE-vitrified blastocysts. The mRNA transcript abundances of the *SOD1* gene were significantly higher in blastocysts derived from SE- than LE-vitrification, although *SOD1* mRNA abundances in both vitrification groups did not differ from those detected in blastocysts derived from fresh non-vitrified blastocysts. No differences in *AQP3*, *CX43*, and *IFNτ* transcript abundances were observed between treatments. However, expanded and hatched blastocysts derived from blastocyst vitrified using the SE protocol showed a clear trend (*p* = 0.07) towards higher *CX43* and *AQP3* gene expression levels compared to expanded and hatched blastocysts vitrified using the LE protocol. When gene expression was compared between blastocyst stages, hatched blastocysts derived from vitrified blastocysts had a higher *CX43* expression and a lower *IFNτ* expression than their expanded counterparts, while no differences between the two stages were observed for the other genes.

## 4. Discussion

To effectively transfer vitrification technology to the field, the procedures used for the warming and transfer of cryopreserved bovine embryos should be kept as simple as possible. The VitTrans device was designed to facilitate the vitrification/warming technique by including an easy one-step in-straw dilution method followed by direct embryo transfer to the uterus [16]. The objective of this study was to investigate the effects of different equilibration times on several post-warming outcome measures in bovine D7 and D8 expanded blastocysts vitrified using the vitrification/in straw-warming VitTrans procedure. Our results indicated that a short equilibration time (3 min) during vitrification improves post-warming survival and the hatching ability of both D7 and D8 expanded blastocysts, whereas lengthening the equilibration time to 12 min does not seem to offer any further benefits. In addition, the hatching rates of D7-blastocysts vitrified by the SE protocol were similar to those recorded for fresh non-vitrified embryos. Several studies have compared equilibration times used in the vitrification of in vitro produced blastocysts of different species [26,27,28,29,30]. In cattle, Do et al. [28] found similar re-expansion (24 h post-warming) and hatching rates (48 h post-warming) when bovine expanded blastocysts were vitrified after a short (3 min) or long equilibration (8 min) time, possibly explained by differences in temperature, equilibration time, and cryodevice used with a three-step warming procedure. Thus, while the short equilibration time tested by Do et al. [28] was similar to ours (3 min at 37 °C), their long equilibration protocol consisted of 8 min at room temperature, which may have resulted in reduced cytotoxicity and osmotic stress [17] and thus minimized differences between the use of their short and long protocol. Consistently, in a study carried out in the dromedary camel, the loading of CPAs at 37 °C for a short exposure time (3 min) led to an outcome comparable to that of original processing at room temperature with a longer exposure time (15 min) [27]. When working at room temperature in humans and mice, different equilibration times were not found to affect post-warming embryo survival [26,29]. However, lengthening the exposure time to the equilibration solution from 4 to 8 min was found to improve the DNA integrity index after the vitrification of murine blastocysts [26,29]. Prior to the vitrification of human blastocysts, 9–10 min of exposure to an equilibration solution improved the outcomes clinical pregnancy, embryo implantation, and live birth rates compared to shorter exposure times [26]. It is noteworthy that in all the previous studies comparing equilibration times in vitrification [26,27,28,29], the warming procedure consisted of successive dilution steps, while here, we report results from an in-straw one-step warming method. 

Different vitrification outcomes have been recently reported after the vitrification of expanded blastocysts using various one-step warming devices and short equilibration times. As we also observed, the one-step in-straw warming/dilution of expanded blastocysts vitrified in fiber plugs returned similar [9] or higher survival rates [21] for D7 blastocysts than D8 blastocysts. However, either lower [9] or higher [15,21] hatching rates were observed at 24 h post-warming when D7 or D8 expanded blastocysts were vitrified compared to our results. Further, the one-step warming of vitrified bovine D7 expanded blastocysts led to higher hatching rates assessed at 48 [15] or 72 h post-warming rather than 24 h post-warming [12,20]. 

When 24 h post-warming outcomes were compared after the vitrification of blastocysts produced after different times of in vitro culture, our results were consistent with those of others. Thus, significantly higher survival, re-expansion, and hatching rates have been described after the vitrification of D7 compared to D8 IVP bovine blastocysts [21,22,31,32], such that cryotolerance diminishes as the length of the embryo culture increases. In the present study, although the hatching ability of D7 blastocysts vitrified/warmed within the SE protocol was comparable that of fresh non-vitrified D7 blastocysts, D8 vitrified/warmed blastocyst gave rise to under half of the hatching yield observed in the fresh control group. Early developing embryos are better at surviving than later embryos. This has been highlighted in prior work in which vitrified/warmed earlier cryopreserved IVP bovine blastocysts returned higher survival, hatching, and pregnancy rates [21,22].

The correct distribution of cells in the ICM and outer TE layer of the blastocyst is crucial for embryo development. However, while it is accepted that a minimal number of embryonic cells is needed to establish pregnancy [33], optimal ICM and TE cell numbers and distributions in the blastocyst remain unclear. Thus, higher ICM cell counts may lead to increased pregnancy rates [34], and an excessive number of cells allocated to the TE may lead to pregnancy abnormalities [35,36]. Here, the TUNEL assay combined with differential staining for ICM and TE cells revealed significantly lower TCN and TE cell numbers and a higher apoptosis rate in vitrified/warmed D7 re-expanded blastocysts compared to fresh ones, while no differences emerged in ICM cell numbers, suggesting that the main site of cryopreservation-related membrane damage was the trophectoderm. Similar [37] or reduced total cell counts have been reported after bovine embryo vitrification [38,39], mainly due to a low cell count in the TE. This effect is consistent with a greater accumulation of lipids in the TE than ICM [40], as cytoplasmic lipid contents appear to be strongly related to the survival of cryopreservation [2]. In contrast, Gomez et al. [41] found that vitrification seemed to exert a detrimental effect on the ICM, while TE cells survived cryopreservation in numbers comparable to those counted in embryos before vitrification. However, we detected no differences in TCN, ICM, and TE cell numbers between D7 blastocysts vitrified after SE and fresh blastocysts, while D7 blastocysts vitrified after LE showed significantly lower TCN and TE cell numbers. This suggests that D7 expanded blastocysts vitrified using our SE protocol suffered less cryodamage or were able to recover from any damage at 24 h post-warming, showing similar hatching rates and embryo quality as those of fresh ones. However, among the D8 embryos subjected to vitrification/warming, TCN, ICM, and TE cell numbers were significantly lower in hatching blastocysts when compared to fresh blastocysts, regardless of the equilibration time. The timing of blastocyst formation is a good marker of embryo quality, with early-cavitating embryos being of better quality than later cavitating embryos in terms of total cell numbers, inner cell mass and trophectoderm cell distributions, and cryosurvival [35,39]. While we still lack reliable blastocyst stage morphological predictors of competence after embryo transfer, it is accepted by many research groups and commercial companies that best pregnancy rates are achieved after the transfer of D7 expanded bovine blastocysts, whether fresh or cryopreserved (reviewed by [42,43]). 

Apoptosis has been frequently used as a marker for embryo quality because high rates of apoptotic cells have been linked to the reduced developmental competence of both in vivo or in vitro produced embryos [44,45,46]. Vitrification requires adequate dehydration and a high viscosity across all blastomeres and blastocele, which is difficult given the characteristics of the blastocyst (multicellularity and the presence of a blastocele with a high water content). This means that vitrification leads to a post-warming increase in apoptosis [47]. Our results revealed that both equilibration solution exposure times induced apoptosis in surviving blastocysts by the time of their re-expansion and hatching. However, while the apoptosis rate for D7 expanded blastocysts vitrified via the VitTrans LE protocol was similar to that reported previously by Morató and Mogas [16], the apoptotic cell rate was significantly higher for the LE than SE protocol or control embryos. This finding suggested that the high toxicity effect of CPAs produced at high temperature can be avoided to some extent by reducing the time of exposure to the cryoprotectant [17]. Moreover, D8 embryos induced higher percentages of apoptotic cells that D7 embryos, which was in agreement with results observed when expanded blastocysts were vitrified/warned using a one-step direct transfer procedure [21].

When genes related to apoptosis were analyzed, a significantly higher abundance of *BCL2L1* transcripts was observed in both expanded and hatched blastocysts derived from the SE protocol when compared to fresh embryos or those vitrified after the long equilibration period, while there were no differences in *BAX* gene expression among treatments. Yang and Rajamahendran [48] related a higher expression level of Bcl-2 to better quality embryos less prone to apoptosis. However, the levels of *BCL2L1* gene expression observed in our study were inconsistent with apoptosis levels assessed by TUNEL in fresh or vitrified D7 blastocysts, suggesting that apoptosis detected by TUNEL is independent of the expression of *BCL2L1* or *BAX* genes, as observed previously [49]. Similarly, the mRNA levels of *SOD1* were upregulated after SE treatment, indicating that a shorter exposure time may reduce oxidative stress by improving the activity of antioxidant enzymes and improving the quality of vitrified/warmed embryos [50]. In addition, a trend—though not significant (*p* = 0.07)—was observed towards greater *CX43* and *AQP3* gene expression in blastocysts subjected to SE compared to LE. In effect, a high expression of *CX43*, a gene related to cell compaction and adhesion [51], has been linked to better quality and more cryotolerant embryos [52]. *AQP3* plays an important role in the transport of cryoprotectants and fluids during the cryopreservation of bovine embryos [53]. The presence of mRNA encoding this protein has been also related to better embryo cryotolerance [54]. While not always significant, the differences in gene expression observed in surviving blastocysts derived from D7 blastocysts vitrified after the SE treatment could be indicative of better embryo quality. In effect, these blastocysts showed an improved hatching ability, together with higher TCN and TE cell numbers, as well as a lower apoptosis rate.

Further experiments on embryo transfer are required to determine if improvements observed in post-warming outcomes of vitrified IVP embryos after a short exposure to the equilibration solution are related to a higher pregnancy rate after in-straw-warming/dilution in field conditions.

## 5. Conclusions

In conclusion, the vitrification of IVP D7 bovine embryos using the in-straw vitrification/warming device with a brief 3 min exposure to the equilibration solution gave rise to post-warming outcomes comparable to those of fresh non-vitrified blastocysts. In addition, our gene expression analysis indicated that the SE treatment could lead to the production of more high-quality blastocysts, promoting the efficiency of embryo transfer. This strategy of shortening the exposure time to the equilibration medium within the in-straw vitrification/warming procedure could have important implications for commercial in vitro embryo transfer programs because it shortens the time needed to vitrify each embryo and simplifies the use of this technique in field conditions. Future experiments are underway to establish the full survival potential of these cryopreserved embryos after their transfer to recipient cows.

## Figures and Tables

**Table 1:** Primers used for reverse transcription-quantitative polymerase chain reaction.

Symbol	Primer Sequences (5′-3′)	Amplicon Size (bp)	GenBank Accession No.
BCL2 associated X apoptosis regulator (*BAX*)	F: ACCAAGAAGCTGAGCGAGTG	116	NM_173894.1
	R: CGGAAAAAGACCTCTCGGGG		
BCL2-like 1 (*BCL2L1*)	F: GAGTTCGGAGGGGTCATGTG	211	NM_001166486.1
	R: TGAGCAGTGCCTTCAGAGAC		
Superoxide dismutase 1 (*SOD1*)	F: ACACAAGGCTGTACCAGTGC	102	NM_174615.2
	R: CACATTGCCCAGGTCTCCAA		
Aquaporin 3 (*AQP3*)	F: GTGGACCCCTACAACAACCC	222	NM_001079794.1
	R: CAGGAGCGGAGAGACAATGG		
Connexin 43 (*CX43*)	F: TGGAATGCAAGAGAGGTTGAAAGAGG	294	NM_174068.2
	R: AACACTCTCCAGAACACATGATCG		
Interferon tau (*IFNτ*)	F: CTGAAGGTTCACCCAGACCC	197	AF238612
	R: GAGTCTGTTCATTCGGGCCA		
Peptidylprolyl isomerase A (*PPIA*)	F: CATACAGGTCCTGGCATCTTGTCC	108	NM_178320.2
	R: CACGTGCTTGCCATCCAACC		
H3.3 histone A (*H3F3A*)	F: CATGGCTCGTACAAAGCAGA	136	NM_001014389.2
	R: ACCAGGCCTGTAACGATGAG		

**Table 2:** Post-warming survival and hatching rates of day seven (D7) and day eight (D8) expanded blastocysts vitrified after shorter or longer exposure to the equilibration solution. Data are shown as mean ± standard error of the mean (SEM).

	Day 7 Blastocysts				Day 8 Blastocysts			
	n	Survival (%) (3 h)	Survival (%) (24 h)	Hatching Rate (%) (24 h)	n	Survival (%) (3 h)	Survival (%) (24 h)	Hatching Rate (%) (24 h)
Control	86	100 ^a,A^	100 ^a,A^	35.9 ± 4.0 ^a,A^	40	100 ^a,A^	100 ^a,A^	50.0 ± 7.0 ^a,B^
SE	86	60.6 ± 1.5 ^b,A^	78.4 ± 2.0 ^b,A^	31.4 ± 3.7 ^a,A^	33	48.6 ± 5.3 ^b,B^	63.0 ± 5.5 ^b,B^	19.9 ± 2.7 ^b,B^
LE	83	57.5 ±4.0 ^b,A^	63.1 ± 2.6 ^c,A^	10.1 ± 2.4 ^b,A^	36	39.4 ± 4.7 ^b,B^	55.3 ± 5.0 ^c,B^	8.1 ± 2.7 ^c,A^

^a,b,c^ Values within columns with different superscripts differ significantly (*p* < 0.05); ^A,B^ Same values within rows with different superscripts differ significantly (*p* < 0.05). Control: fresh non-vitrified expanded blastocysts; SE: expanded blastocysts vitrified after a short equilibration time (3 min); LE: expanded blastocysts vitrified after a long equilibration time (12 min).

**Table 3:** TCN, ICM, and TE cell numbers and rate of apoptotic cells in warmed D7 and D8 expanded bovine blastocysts vitrified after a short or long exposure time to the equilibration solution. Data are shown as mean ± SEM.

	**Day 7 Blastocysts**								
		**TCN**		**ICM Cell Number**		**TE Cell Number**		**AR**	
	n	Expanded	Hatched	Expanded	Hatched	Expanded	Hatched	Expanded	Hatched
Control	30	140.3 ± 8.6 ^a,A^	189.8 ± 4.4 ^a,B^	24.4 ± 1.6 ^a,A^	38.6 ± 1.6 ^a,B^	115.9 ± 7.9 ^a,A^	151.3 ± 4.1 ^a,B^	3.7 ± 0.4 ^a^	4.7 ± 0.7 ^a^
SE	23	110.0±2.7 ^b,A^	195.2 ± 3.5 ^a,B^	22.0 ± 2,1 ^a,A^	35.1 ± 1.5 ^a,B^	87.1 ± 2.3 ^b,A^	160.1 ± 3.1 ^a,B^	11.5 ± 1.1 ^b^	9.1 ± 0.9 ^b^
LE	21	113.2 ± 4.5 ^b,A^	170.6 ± 1.4 ^b,B^	23.3 ± 1.3 ^a,A^	33.2 ± 3.3 ^a,B^	89.9 ± 4.3 ^b,A^	135.4 ± 2.8 ^b,B^	15.2 ± 0.3 ^c^	13.6 ± 1.2 ^c^
	**Day 8 Blastocysts**								
		**TCN**		**ICM Cell Number**		**TE Cell Number**		**AR**	
	n	Expanded	Hatched	Expanded	Hatched	Expanded	Hatched	Expanded	Hatched
Control	40	125.3 ± 5.4 ^a,A^	206.8 ± 12 ^a,B^	29.2 ± 1.9 ^a,A^	43.1 ± 1.2 ^a,B^	96.0 ± 4.5 ^a,A^	163.6 ± 5.6 ^a,B^	5.6 ± 0.4 ^a^	4.6 ± 0.7 ^a^
SE	21	128.1 ± 2.8 ^a,A^	169.7 ± 5.6 ^b,B^	22.2 ± 1.0 ^b,A^	32.0 ± 1.2 ^b,B^	105.9 ± 2.8 ^a,A^	137.8 ± 4.0 ^b,B^	15.1±0.6 ^b^	12.7 ± 0.8 ^b^
LE	20	108.4 ± 1.3 ^b,A^	154.6 ± 2.1 ^b,B^	19.7 ± 0.8 ^b,A^	29.0 ± 2.0 ^b,B^	88.7 ± 1.3 ^b,A^	125.0 ± 2.7 ^b,B^	25.1 ± 1.5 ^c^	23.2 ± 2.1 ^c^

^a,b,c^ Values within columns with different superscripts differ significantly (*p* < 0.05); ^A,B^ Values within rows with different superscripts differ significantly (*p* < 0.05). TCN: total cell number; ICM: inner cell mass; TE: trophectoderm; AR: apoptosis rate. Expanded: expanded blastocyst; Hatched: hatching/hatched blastocyst. Control: fresh non-vitrified expanded blastocysts; SE: expanded blastocysts vitrified after a short equilibration period (3 min); LE: expanded blastocysts vitrified after a long equilibration period (12 min).

## Data Availability

Data is contained within the article.
